# Automated Deep Learning–based Segmentation of the Dentate Nucleus Using Quantitative Susceptibility Mapping MRI

**DOI:** 10.1148/ryai.240478

**Published:** 2025-08-06

**Authors:** Diogo H. Shiraishi, Susmita Saha, Isaac M. Adanyeguh, Sirio Cocozza, Louise A. Corben, Andreas Deistung, Martin B. Delatycki, Imis Dogan, William Gaetz, Nellie Georgiou-Karistianis, Simon Graf, Marina Grisoli, Pierre-Gilles Henry, Gustavo M. Jarola, James M. Joers, Christian Langkammer, Christophe Lenglet, Jiakun Li, Camila C. Lobo, Eric F. Lock, David R. Lynch, Thomas H. Mareci, Alberto R. M. Martinez, Serena Monti, Anna Nigri, Massimo Pandolfo, Kathrin Reetz, Timothy P. Roberts, Sandro Romanzetti, David A. Rudko, Alessandra Scaravilli, Jörg B. Schulz, S. H. Subramony, Dagmar Timmann, Marcondes C. França, Ian H. Harding, Thiago J. R. Rezende, for the TRACK-FA Neuroimaging Consortium

**Affiliations:** ^1^Department of Neurology, School of Medical Sciences, University of Campinas (Unicamp), Rua Vital Brasil, 89-99, Cidade Universitária “Zeferino Vaz”, Campinas, São Paulo, Brazil 13083-888; ^2^School of Psychological Sciences, The Turner Institute for Brain and Mental Health, Monash University, Clayton, Australia; ^3^Department of Neuroscience, School of Translational Medicine, Monash University, Melbourne, Australia; ^4^Center for Magnetic Resonance Research, Department of Radiology, University of Minnesota, Minneapolis, Minn; ^5^Department of Advanced Biomedical Sciences, University of Naples Federico II, Naples, Italy; ^6^Bruce Lefroy Centre for Genetic Health Research, Murdoch Children’s Research Institute, Parkville, Australia; ^7^Department of Paediatrics, University of Melbourne, Parkville, Australia; ^8^University Clinic and Outpatient Clinic for Radiology, Department for Radiation Medicine, University Hospital Halle (Saale), University Medicine Halle, Halle (Saale), Germany; ^9^Halle MR Imaging Core Facility, Medical Faculty, Martin-Luther-University Halle-Wittenberg, Halle (Saale), Germany; ^10^Department of Neurology, RWTH Aachen University, Aachen, Germany; ^11^JARA-BRAIN Institute Molecular Neuroscience and Neuroimaging, Forschungszentrum Jülich GmbH and RWTH Aachen University, Aachen, Germany; ^12^Department of Radiology, Children’s Hospital of Philadelphia, Philadelphia, Pa; ^13^Department of Neuroradiology, Fondazione IRCCS Istituto Neurologico Carlo Besta, Milan, Italy; ^14^Department of Neurology, Medical University of Graz, Graz, Austria; ^15^Division of Biostatistics and Health Data Science, School of Public Health, University of Minnesota, Minneapolis, Minn; ^16^Department of Neurology, Children’s Hospital of Philadelphia, Philadelphia, Pa; ^17^Department of Biochemistry and Molecular Biology, University of Florida, Gainesville, Fla; ^18^Institute of Biostructures and Bioimaging, Italian National Research Council, Naples, Italy; ^19^Department of Neurology and Neurosurgery, McGill University, Montreal, Canada; ^20^McConnell Brain Imaging Centre, Montreal Neurological Institute and Hospital, Montreal, Canada; ^21^Department of Biomedical Engineering, McGill University, Montreal, Canada; ^22^Department of Neurology and the Fixel Institute for Neurological Diseases, University of Florida, Gainesville, Fla; ^23^Department of Neurology and Center for Translational and Behavioral Neuroscience (C-TNBS), Essen University Hospital, University of Duisburg-Essen, Essen, Germany; ^24^QIMR Berghofer Medical Research Institute, Brisbane, Australia; ^25^School of Translational Medicine, Monash University, Melbourne, Australia; ^26^Paulo Gontijo Institute, São Paulo, Brazil

**Keywords:** MR Imaging, Brain/Brain Stem, Segmentation, Convolutional Neural Network, Supervised Learning, Computer Applications–3D, Volume Analysis, Image Postprocessing

## Abstract

**Purpose:**

To develop a dentate nucleus (DN) segmentation tool using deep learning applied to brain MRI–based quantitative susceptibility mapping (QSM) images.

**Materials and Methods:**

Brain QSM images from healthy controls and individuals with cerebellar ataxia or multiple sclerosis were collected from nine different datasets (2016–2023) worldwide for this retrospective study (ClinicalTrials.gov identifier: NCT04349514). Manual delineation of the DN was performed by experienced raters. Automated segmentation performance was evaluated against manual reference segmentations following training with several deep learning architectures. A two-step approach was used, consisting of a localization model followed by DN segmentation. Performance metrics included intraclass correlation coefficient (ICC), Dice score, and Pearson correlation coefficient.

**Results:**

The training and testing datasets comprised 328 individuals (age range, 11–64 years; 171 female individuals), including 141 healthy individuals and 187 with cerebellar ataxia or multiple sclerosis. The manual tracing protocol produced reference standards with high intrarater (average ICC, 0.91) and interrater reliability (average ICC, 0.78). Initial deep learning architecture exploration indicated that the nnU-Net framework performed best. The two-step localization plus segmentation pipeline achieved a Dice score of 0.90 ± 0.03 (SD) and 0.89 ± 0.04 for left and right DN segmentation, respectively. In external testing, the proposed algorithm outperformed the current leading automated tool (mean Dice scores for left and right DN, 0.86 ± 0.04 vs 0.57 ± 0.22 [*P* < .001]; 0.84 ± 0.07 vs 0.58 ± 0.24 [*P* < .001]). The model demonstrated generalizability across datasets unseen during the training step, with automated segmentations showing high correlation with manual annotations (left DN: *r =* 0.74 [*P* < .001]; right DN: *r =* 0.48 [*P* = .03]).

**Conclusion:**

The proposed model accurately and efficiently segmented the DN from brain QSM images. The model is publicly available *(https://github.com/art2mri/DentateSeg)*.

**Keywords:** MR Imaging, Brain/Brain Stem, Segmentation, Convolutional Neural Network, Supervised Learning, Computer Applications–3D, Volume Analysis, Image Postprocessing

ClinicalTrials.gov registration no. NCT04349514

*Supplemental material is available for this article.*

© The Author(s) 2026. Published by the Radiological Society of North America under a CC BY 4.0 license.

## Graphical abstract

**Figure:**
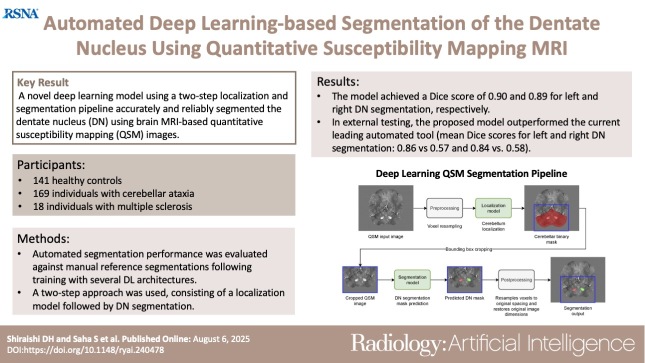

**Summary**

A deep learning model using a two-step localization and segmentation pipeline accurately and reliably segmented the dentate nucleus using brain MRI–based quantitative susceptibility mapping images.

**Key Points**

■ A diverse dataset of 328 individuals (141 healthy, 169 with cerebellar ataxia, and 18 with multiple sclerosis) collected across 10 sites using varied MRI scanners, protocols, and quantitative susceptibility mapping methods was used to develop and evaluate a deep learning model for automated segmentation of the dentate nucleus (DN).
■ The proposed segmentation pipeline achieved a high Dice score (0.90 ± 0.03 [SD] for the left DN and 0.89 ± 0.04 for the right DN), outperforming the only alternative publicly available solution (MRICloud) and demonstrating robustness and generalizability across external datasets.

## Introduction

The dentate nuclei (DN) are the largest of the deep cerebellar nuclei and are the primary efferent stations of the human cerebellum. The DN are primarily innervated by Purkinje cells of the lateral hemispheres of the cerebellar cortex and give rise to the major ascending cerebellar output pathways, including the dentatorubral and dentato-thalamo-cortical tracts (1). The DN are divided into motor and nonmotor functional subregions with distinct profiles of disynaptic axonal innervation to cerebral cortices (1). Abnormalities in the DN may therefore contribute to disruption in large-scale brain networks involved in a broad array of behavioral deficits (1). Furthermore, neuropathologic studies have provided evidence that the DN play a central role in the pathogenesis of several cerebellar diseases, particularly in inherited cerebellar ataxias (1).

Despite the established importance of the DN in cerebrocerebellar loops and growing evidence of involvement in brain diseases, direct in vivo quantitative investigations of DN structure using neuroimaging in humans are scarce (2–5). Such investigations have been particularly challenging due to the tissue properties of DN that make them invisible or poorly defined using standard MRI sequences (T1-weighted and T2-weighted images). Susceptibility-weighted MRI offers a limited solution, allowing for visualization of the DN due to their high iron content (6). However, susceptibility-weighted MRI is a qualitative technique that is primarily used for the clinical detection of vascular abnormalities and microangiopathies (6). Although useful, susceptibility-weighted MRI has several limitations, including its nonquantitative nature and distortion of tissue boundaries due to blooming effects and nonlocal phase contributions of the iron deposits in the tissues. These limitations have largely been overcome through the development of quantitative susceptibility mapping (QSM) (7). QSM allows for more precise mapping of the anatomy and a more direct link to the underlying iron concentration (6), providing opportunities for direct quantitative evaluation of DN structure and microstructure in clinical populations (2,5).

Although QSM holds great promise for quantifying and tracking DN changes in disease, a major roadblock to date in undertaking large-scale and reliable QSM investigations of the DN in patient cohorts has been the reliance on manual hand-drawn segmentations. To overcome this limitation, the MRICloud toolkit (8) provided the first and, to our knowledge, the only currently publicly available automated DN segmentation tool. MRICloud has limitations in accuracy and generalizability (9) and is based on a multiatlas approach, a technique which has largely been superseded by contemporary deep learning methods. In this study, we aimed to develop a fully automated DN segmentation tool based on a deep learning approach, using a large, diverse, and high-quality dataset of brain MRI–based QSM images. Hosted in a public repository, the model is available for use and testing in observational, natural history, and treatment trials for biomarker discovery.

## Materials and Methods

### Data

The institutional review board for each project data source or site approved the use or ethics waiver for this retrospective study, and all individuals provided written informed consent before original data collection (Appendix S1) (ClinicalTrials.gov identifier: NCT04349514). The TRACK-FA steering committee approved the data use.

**Figure 1:**
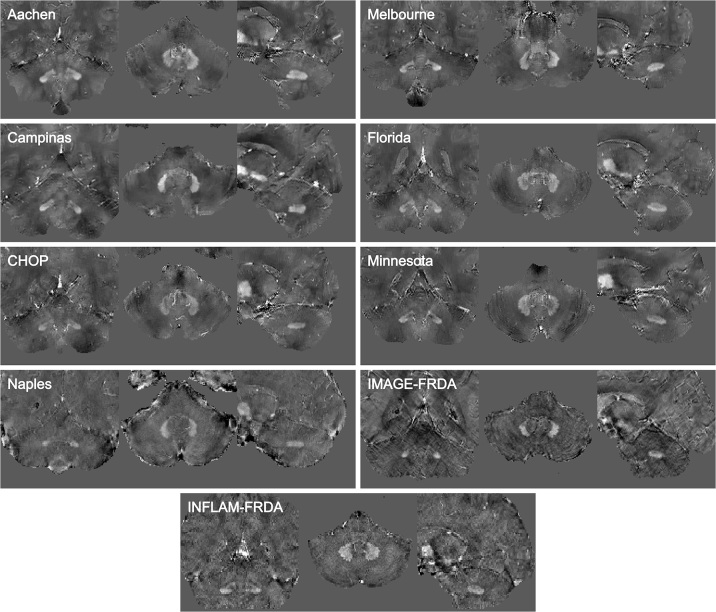
MR images show samples for each dataset. The images were randomly selected and cropped focusing on the cerebellum. Voxel intensities were normalized using z score to improve visualization. No contrast agents were used. Aachen: images in a 32-year-old male patient with Friedreich ataxia (FRDA), from RWTH Aachen University. Campinas: images in a 32-year-old female patient with FRDA, from the University of Campinas. CHOP: images in a 15-year-old female patient with FRDA, from Children’s Hospital of Philadelphia. Naples: images in a 23-year-old male patient with FRDA, from the University of Naples “Federico II”.IMAGE-FRDA (5,32): images in a 32-year-old female healthy control. Melbourne: images in a 23-year-old female patient with FRDA, from Monash University and Murdoch Children’s Research Institute. Florida: images in an 18-year-old female patient with FRDA, from the University of Florida. Minnesota: images in a 28-year-old female healthy control, from the University of Minnesota. INFLAM-FRDA (11): images in a 20-year-old female healthy control.

Multiecho gradient-recalled echo MRI data were acquired using four different MRI protocols (5,10,11) implemented across 10 imaging centers using 3-T Philips or Siemens MRI scanners (Table 1, Fig 1). The collected research data were de-identified at each source, ensuring adherence to a data pipeline free from personal health information. The dataset included a total of 132 healthy individuals as controls, 154 individuals with Friedreich ataxia (FRDA), and 16 individuals with multiple sclerosis (MS). Follow-up scans at 12 months were available from 55 of these individuals. Images of individuals with FRDA or MS were selected to account for anatomic variability in DN caused by neurodegeneration. Healthy individuals had no history of neurologic disease, individuals with FRDA had molecular confirmation, and individuals with MS were diagnosed according to the 2010 McDonald criteria.

**Table 1:** Acquisition Protocols for Each Dataset

Dataset	Scanner	Sequence	TR (msec)	TE1 (msec)	ΔTE(msec)	No. of Echoes	FOV (mm)	Image Matrix (Voxels)	Voxel Size (mm)	Acquisition Time
Aachen	3-T Siemens Prisma	GRE	27	3.7	6	4	220 × 220 × 176	208 × 256 × 176	0.86 iso	7 min 22 sec
Campinas	3-T Philips Ingenia	GRE	27	3.7	6	4	220 × 220 × 176	208 × 256 × 176	0.86 iso	7 min 22 sec
CHOP	3-T Siemens Prisma	GRE	27	3.7	6	4	220 × 220 × 176	208 × 256 × 176	0.86 iso	7 min 22 sec
Florida	3-T Siemens Prisma	GRE	27	3.7	6	4	220 × 220 × 176	208 × 256 × 176	0.86 iso	7 min 22 sec
Melbourne	3-T Siemens Skyra	GRE	27	3.7	6	4	220 × 220 × 176	208 × 256 × 176	0.86 iso	7 min 22 sec
Minnesota	3-T Siemens Prisma	GRE	27	3.7	6	4	220 × 220 × 176	208 × 256 × 176	0.86 iso	7 min 22 sec
Naples	3-T Siemens Magnetom Trio	GRE	32	7.38	14.76	2	230 × 194 × 160	378 × 448 × 160	0.51 × 0.51 × 1.0	9 min 53 sec
IMAGE-FRDA (5,32)	3-T Siemens Skyra	GRE	30	7.38	14.76	2	230 × 230, 160 axial sections	232 × 256 × 160	0.90 iso	11 min 30 sec
INFLAM-FRDA (11)	3-T Siemens Biograph	GRE	31	5.70	5.27	5	230 × 230, 104 axial sections	384 × 384 × 104	0.60 × 0.60 × 1.2	6 min 50 sec

Note.—Aachen = RWTH Aachen University, Campinas = University of Campinas, CHOP = Children’s Hospital of Philadelphia, Florida = University of Florida, FOV = field of view, GRE = gradient-recalled echo, iso = isotropic, Melbourne = Monash University and Murdoch Children’s Research Institute, Minnesota = University of Minnesota, Naples = University of Naples “Federico II,” TE = echo time, TE1 = first echo time, TR = repetition time.

The QSM images were reconstructed using the JHU/KKI QSM (8) and STI Suite *(https://people.eecs.berkeley.edu/~chunlei.liu/software.html)* toolboxes, using Laplacian unwrapping to overcome phase aliasing, V-SHARP for background field removal (12), and MEDI (13) or iLSQR (14) for field-to-susceptibility reconstruction.

**Figure 2:**
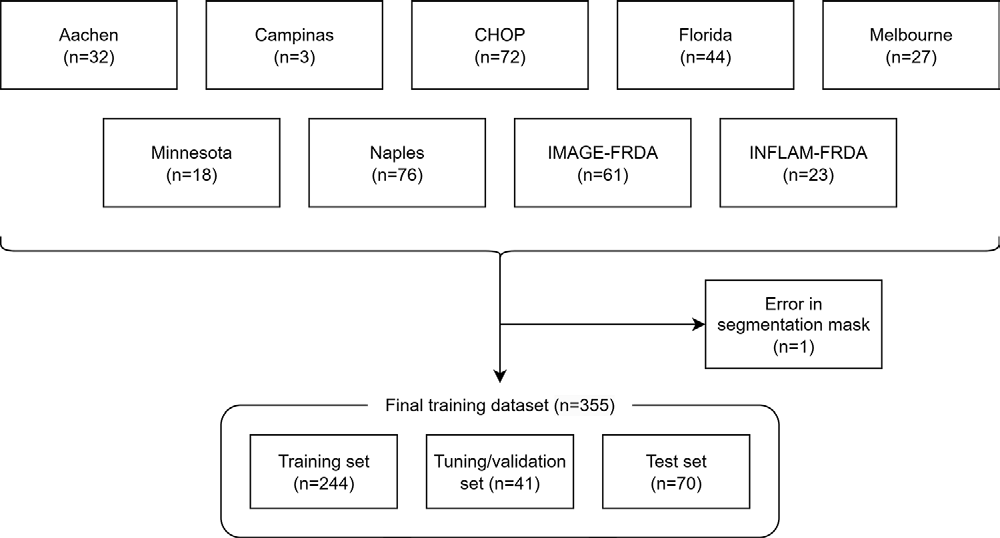
Diagram details the inclusion of image datasets into the study. Details for IMAGE-FRDA dataset are in references 5 and 32; details for INFLAM-FRDA are in reference 11. Aachen = RWTH Aachen University, Campinas = University of Campinas, CHOP = Children’s Hospital of Philadelphia, Florida = University of Florida, FRDA = Friedreich ataxia, Melbourne = Monash University and Murdoch Children’s Research Institute, Minnesota = University of Minnesota, Naples = University of Naples “Federico II.”

**Figure 3:**
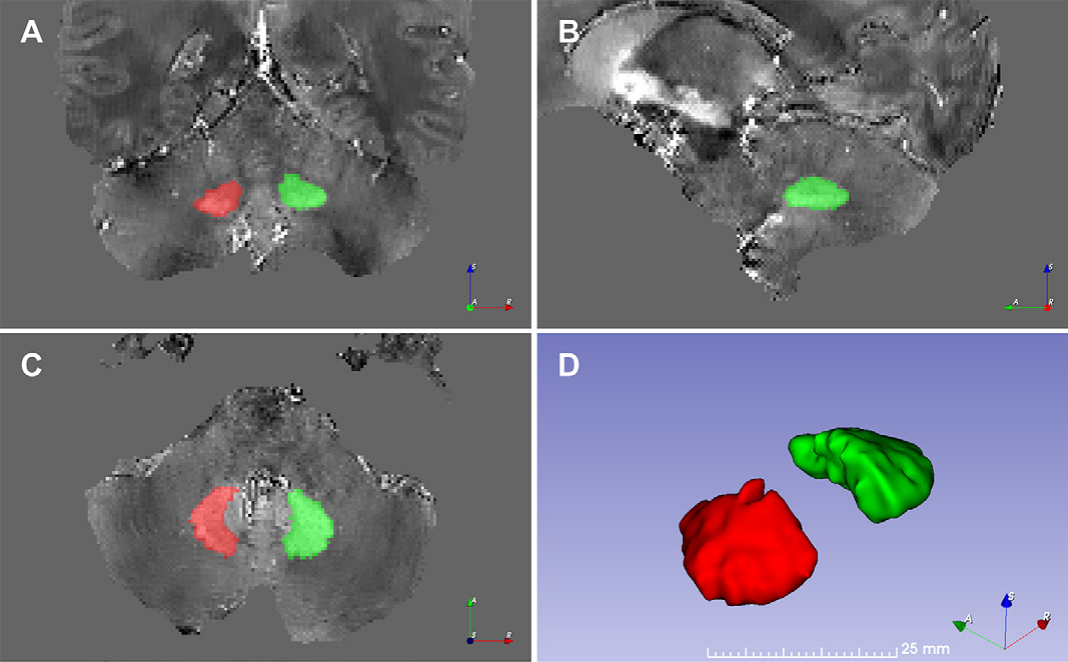
Manually segmented mask MR images in a 41-year-old female healthy control delineate the left dentate nucleus in red and the right dentate nucleus in green from a sample quantitative susceptibility mapping dataset. **(A)** Coronal, **(B)** sagittal, and **(C)** axial MR images, as well as a **(D)** shaded-surface display are shown. No contrast agents were used.

To train the deep learning (DL) models, we divided the data into 70%, 10%, and 20% proportions for training, tuning, and internal testing sets, respectively (Fig 2). We applied stratified sampling, creating groups that maintain the relative distributions by MRI acquisition center, while keeping images belonging to an individual in just one set, minimizing the risk of data leakage. To mitigate potential biases, three independent neuroradiologists with 7 (S.S.), 10 (S.C.), and 10 (I.H.H.) years of experience, blinded to individual data and diagnosis, provided annotated references (Fig 3) (protocol in Appendix S1).

To compare our results with MRICloud, we processed the test images through their web-based service (8), using its susceptibility multiatlas tool. MRICloud jobs for the segmentation model architecture comparison section were executed from January 5, 2023, to January 7, 2023, and from March 9, 2024, to March 31, 2024, for the external testing datasets.

### DL Architectures

Convolutional neural network architectures are typically used to process image data. For segmentation tasks, several studies rely on U-Nets (15) and their three-dimensional variation (16), both based on convolutional neural networks. Numerous variations of these architectures have been reported in the literature, such as the one provided by the nnU-Net (17) framework. More recently, new architectures based on transformers (18) were introduced, which arose in the language sequence models domain, including UNETR (19) and Swin UNETR (20). In this work, we applied and contrasted the performance of three DL architectures (Appendix S1) to develop an optimal approach for automated DN parcellation. To improve model generalization capacity, we applied data augmentation techniques such as flipping, rotation, scaling, elastic deformation, and intensity scale and shift operations (21).

### DL Segmentation Pipeline

**Figure 4:**
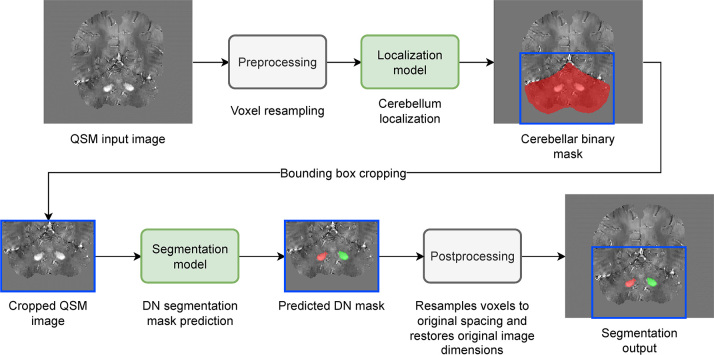
Graphic shows the deep learning quantitative susceptibility mapping (QSM) segmentation pipeline. DN = dentate nucleus.

The DL segmentation pipeline is presented in Figure 4. First, the QSM image was resampled to a common isotropic voxel spacing (0.86 mm), consistent with the original resolution of most of the datasets. The second step was the application of the localization model, which is a DL network designed to locate the cerebellum within a three-dimensional MRI dataset (Appendix S1). The third step was the segmentation stage. The output of this model was the mask with left and right DN labels. Subsequently, another resampling process provides the predicted DN segmentation mask in the original voxel spacing and in the same dimensions as the input image. Modeling was performed using Python version 3.10.11 (Python Software Foundation), PyTorch version 2.2.2, MONAI version 1.0.1, TorchIO version 0.18.85, scikit-learn version 1.0.2, and nnUnetv2 version 2.3.1 (see Appendix S1).

### External Testing

Twenty-six unseen individuals from four new imaging sites (institutional review board approvals detailed in Appendix S1) contributed a total of 38 datasets for external testing (Tables S1–S4). Site 13B provided 17 images using a specific imaging protocol that restricted the field of view to the DN only (Fig S6), unsuitable for MRICloud due to the lack of T1-weighted image pairing with the QSM (Appendix S1). QSM maps were derived using both the JHU/KKI QSM version 3.0 (8) and SEPIA version 1.2.2.6 (22) toolboxes, resulting in two images per individual for site 10B and site 11B datasets. Three reacquired site 10B anisotropic QSM images processed with SEPIA were added. An annotator (S.S.) manually segmented the DN, and the data were processed through our model and MRICloud to compare their performances. For SEPIA processing, three-dimensional best path was used for phase unwrapping and MEDI nonlinear fit for echo-phase combination, Laplacian boundary value approach was used for background magnetic field removal, and QSM images were obtained using STreaking Artifact Reduction for QSM (23).

### Biologic Validation

Further validation of the model in an applied setting was undertaken by replicating previously reported associations between age, DN volume, and DN susceptibility. Pearson correlations were applied for this analysis.

### Statistical Analysis

To assess network performance, Dice score is a widely used metric for the evaluation of segmentation tasks, representing a measure of overlap between two binary masks. We computed the Hausdorff distance and average Hausdorff distance, relevant for boundary quality analysis. Intersection over union or Jaccard index was evaluated. Volume similarity measured the quantitative volume agreement, ignoring shape, and position. The Kolmogorov-Smirnov test was employed to assess normality in continuous variables. To compare metrics between models, we used the Wilcoxon signed rank test. Pearson correlation coefficients were chosen to evaluate relationships within variables. We measured the reliability of measurements using the intraclass correlation coefficient (ICC) (24). Statistical analyses were conducted using Python version 3.10.8, R version 4.1.1, and SPSS version 27.0.1.0. *P* values < .05 (Bonferroni corrected) were considered statistically significant.

### Data and Code Availability

Data generated or analyzed during the study are available from the corresponding author by request. TRACK-FA (N.G.K., coordinator principal investigator) and Enigma-Ataxia (I.H.H., principal investigator) data analyzed during the study were provided by a third party.

The model is publicly available *(https://github.com/art2mri/DentateSeg)*.

## Results

### Individual Characteristics

Demographic characteristics of individuals are shown in Table 2 (training data) and Tables S2 and S4 (external testing data). The total study dataset included 328 individuals (302 in training and 26 in external testing) aged between 11 and 64 years old, with 157 (47.9%) male and 171 (52.1%) female individuals. Among all study individuals, 141 (43.0%) were healthy, 169 (51.5%) had FRDA, and 18 (5.5%) had MS.

**Table 2:** Individual Demographics for Each Dataset

Parameters	Aachen, Campinas, CHOP, Florida, Melbourne, Minnesota		Naples			IMAGE-FRDA (5,32)		INFLAM-FRDA (11)	
	Controls	FRDA	Controls	FRDA	MS	Controls	FRDA	Controls	FRDA
Individuals	44	98	48	12	16	31	30	9	14
Children (<18 years)	14	31	0	3	0	0	0	0	0
Age (y)									
Male individuals	23.8 ± 8.4 (11–40)	24.5 ± 9.0 (13–42)	35.7 ± 10.9 (19–59)	37.9 ± 10.6 (23–49)	46.0 ± 9.6 (35–57)	39.3 ± 12.0 (23–60)	36.9 ± 11.4 (23–55)	26.0 ± 5.7 (19–32)	28.1 ± 7.7 (18–42)
Female individuals	23.3 ± 7.1 (14–41)	23.3 ± 8.5 (12–42)	39.1 ± 13.7 (19–64)	29.9 ± 17.8 (14–62)	47.2 ± 3.1 (43–53)	35.9 ± 14.4 (19–62)	34.1 ± 13.6 (18–56)	29.4 ± 7.4 (20–40)	27.2 ± 7.9 (18–37)
Male-to-female ratio	24:20	49:49	19:29	4:8	5:11	16:15	17:13	4:5	9:5
Disease severity	NA	SARA 14.1 ± 6.1	NA	SARA 15.5 ± 5.4	NA	NA	FARS 79.6 ± 27.7	NA	mFARS 47.0 ± 13.9

Note.—Unless otherwise noted, data are numbers or means ± SDs, with ranges in parentheses. Aachen = RWTH Aachen University, Campinas = University of Campinas, CHOP = Children’s Hospital of Philadelphia, FARS = Friedreich Ataxia Rating Scale, Florida = University of Florida, FRDA = Friedreich ataxia, Melbourne = Monash University and Murdoch Children’s Research Institute, mFARS = modified Friedreich Ataxia Rating Scale, Minnesota = University of Minnesota, MS = multiple sclerosis, NA = not applicable, Naples = University of Naples “Federico II,” SARA = Scale for the Assessment and Rating of Ataxia.

### Intrarater and Interrater Reproducibility Results

All raters demonstrated good to excellent intrarater reliability (ICC > 0.8, Table S5) and moderate to good interrater agreement (ICC > 0.5 for moderate and ICC > 0.75 for good) when evaluated in pairs (Table S6). The mean interrater ICC was 0.76 (95% CI: 0.54, 0.90) for the left DN (LDN) and 0.67 (95% CI: 0.17, 0.88) for the right DN (RDN). The Dice score and Hausdorff distance additionally support strong reliability for the evaluation of segmentation overlapping and annotation deviations (Tables S5, S6). The volume similarity metric, comparing the segmented volumes (in cubic millimeters), indicates that absolute differences within and between raters are marginal (Table S5, S6).

### Final Segmentation Model Outputs

**Figure 5:**
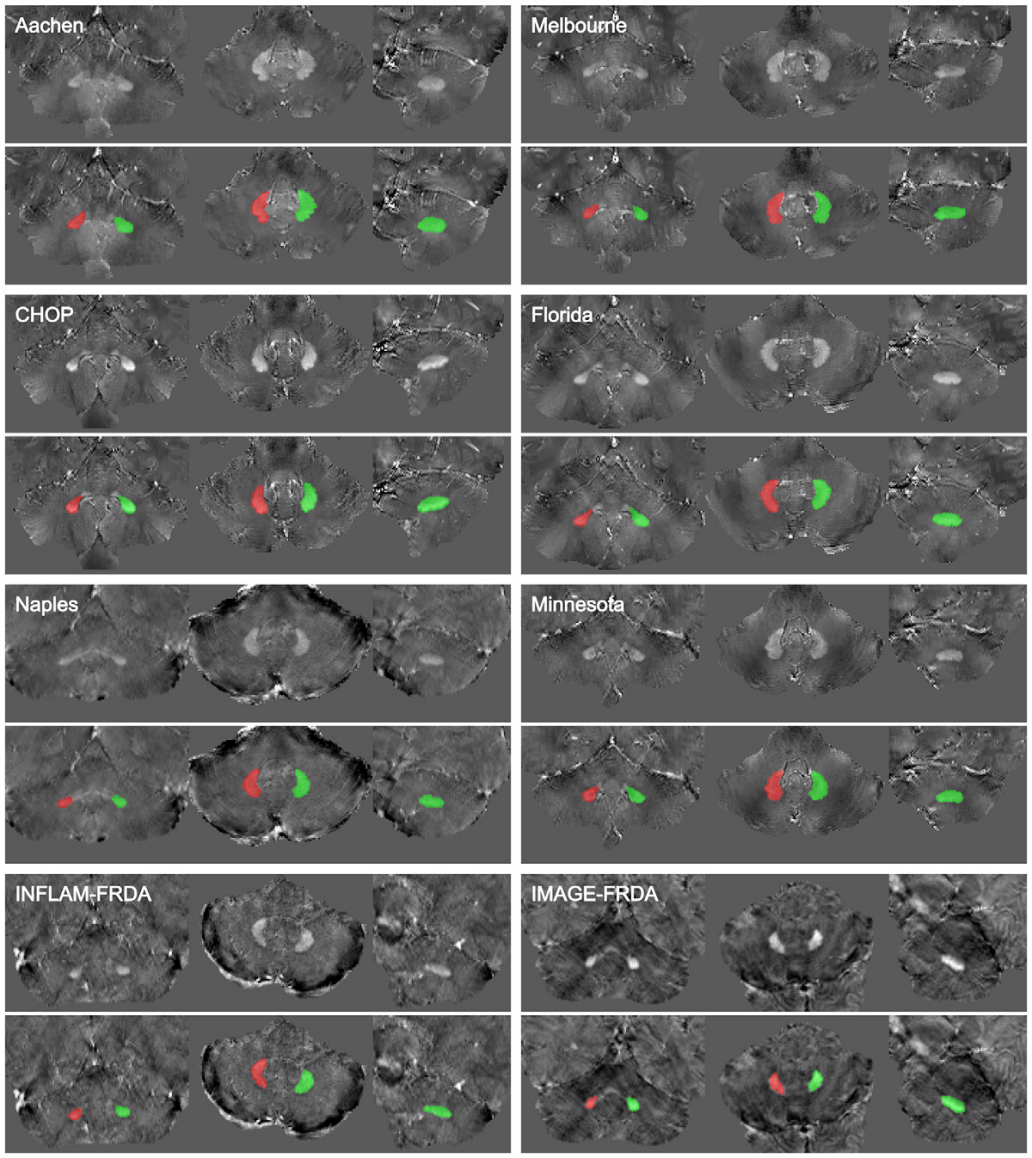
MR images show an overview of pipeline prediction examples for datasets of each center. Quantitative susceptibility mapping images without (top) and with the predicted segmentation mask as overlay (bottom) are presented in the coronal, axial, and sagittal planes, respectively. The left dentate nucleus is depicted in red, and the right dentate nucleus is in green. No contrast agents were used. Aachen: images in a 25-year-old male patient with Friedreich ataxia (FRDA), from RWTH Aachen University. CHOP: images in a 28-year-old female patient with FRDA, from the Children’s Hospital of Philadelphia. Florida: images in a 25-year-old male patient with FRDA, from the University of Florida. Melbourne: images in an 18-year-old male patient with FRDA, from Monash University and Murdoch Children’s Research Institute. Minnesota: images in a 23-year-old female patient with FRDA, from the University of Minnesota. Naples: images in a 38-year-old male patient with FRDA, from the University of Naples “Federico II”.IMAGE-FRDA (5,32): images in a 43-year-old female patient with FRDA. INFLAM-FRDA (11): images in a 21-year-old female patient with FRDA.

The localization model showed a high Dice score (0.92 ± 0.03 [SD]) and runs in less than 5 seconds on CPU-only hardware and provides the model with as much spatial context as possible while considering computational resource constraints. The final trained model (nnU-Net) performed well on the test set (Table 3, Figs S1–S4) for both healthy individuals and those with FRDA or MS (mean Dice, 0.90 ± 0.03 [LDN] and 0.89 ± 0.04 [RDN]). Samples of predicted segmentation masks for each center are shown in Figure 5. The predicted DN volumes highly correlated with the volumes of the reference standard annotations (Fig S3).

**Table 3:** Trained nnU-Net Final Model Performance Metrics for Left Dentate Nucleus and Right Dentate Nucleus on the Complete Test Set

Group	Dice Score		Jaccard Index		Hausdorff Distance		Average Hausdorff Distance		Volume Similarity	
	LDN	RDN	LDN	RDN	LDN	RDN	LDN	RDN	LDN	RDN
All individuals	0.90 ± 0.03	0.89 ± 0.04	0.82 ± 0.05	0.81 ± 0.06	3.12 ± 1.82	4.02 ± 4.57	0.12 ± 0.06	0.14 ± 0.14	0.94 ± 0.04	0.94 ± 0.05
Controls	0.89 ± 0.03	0.89 ± 0.04	0.81 ± 0.05	0.81 ± 0.07	3.41 ± 1.92	3.85 ± 2.05	0.13 ± 0.07	0.13 ± 0.07	0.95 ± 0.05	0.95 ± 0.06
Patients (with either FRDA or MS)	0.90 ± 0.03	0.90 ± 0.03	0.82 ± 0.05	0.81 ± 0.05	2.93 ± 1.78	4.14 ± 5.75	0.11 ± 0.05	0.14 ± 0.18	0.94 ± 0.04	0.94 ± 0.04

Note.—All data are means ± SDs. DN = dentate nucleus, FRDA = Friedreich ataxia, LDN = left DN, MS = multiple sclerosis, RDN = right DN.

### External Testing

Our model performed satisfactorily on an external dataset of 21 images from three different sites, achieving Dice scores of 0.86 ± 0.04 for LDN and 0.84 ± 0.07 for RDN. In contrast, MRICloud demonstrated significantly lower performance for both LDN (Dice, 0.57 ± 0.22; *P* < .001) and RDN (Dice, 0.58 ± 0.24; *P* < .001). Additionally, Pearson correlation coefficients between volumes predicted by the DL model (LDN: *r =* 0.74 [*P* < .001]; RDN: *r =* 0.48 [*P *= .03]) and those manually traced were significant for both DN sides, a result not observed with MRICloud outcomes (LDN: *r =* 0.42 [*P *= .06]; RDN: *r =* 0.19 [*P *= .42]). High Dice scores were observed on the site 13B dataset (LDN: 0.90 ± 0.02; RDN: 0.90 ± 0.02). Interestingly, our model showed consistency across all external datasets when considering the individual QSM subsets per acquisition center. Furthermore, our segmentation pipeline demonstrated robust performance when evaluating susceptibility maps generated by the alternative QSM reconstruction method (STreaking Artifact Reduction for QSM, External Testing section in the Materials and Methods) (Tables 4, S8).

**Table 4:** External Testing Dice Scores for Both Left and Right Dentate Nucleus Comparing Results from MRICloud and Our Proposed DL Model

Dataset	Voxel	QSM Reconstruction	No. of Individuals	No. of Images	MRICloud		Our Model	
					LDN	RDN	LDN	RDN
Site 10B*	Anisotropic	Star-QSM	4	3	0.51 ± 0.12	0.49 ± 0.21	0.83 ± 0.01	0.74 ± 0.07
	Isotropic	MEDI	4	4	0.54 ± 0.30	0.54 ± 0.31	0.90 ± 0.02	0.89 ± 0.02
	Isotropic	Star-QSM	4	4	0.48 ± 0.11	0.52 ± 0.22	0.86 ± 0.04	0.83 ± 0.05
Site 11B	Anisotropic	Star-QSM	2	2	0.25 ± 0.35	0.30 ± 0.42	0.84 ± 0.00†	0.79 ± 0.10
Site 12B*	Isotropic	MEDI	4	4	0.70 ± 0.12	0.71 ± 0.07	0.89 ± 0.01	0.88 ± 0.02
	Isotropic	Star-QSM	4	4	0.76 ± 0.03	0.77 ± 0.03	0.84 ± 0.05	0.87 ± 0.03
Site 13B	Isotropic	MEDI	16	17	NA	NA	0.90 ± 0.02	0.90 ± 0.02
Mean	…	…	…	…	0.57 ± 0.22	0.58 ± 0.24	0.86 ± 0.04	0.84 ± 0.07

Note.—Unless otherwise noted, data are means ± sds. Data from site 13B could not be processed in MRICloud. DN = dentate nucleus, LDN = left DN, QSM = quantitative susceptibility mapping, RDN = right DN, Star-QSM = STreaking Artifact Reduction for QSM.

* All testing used the same individuals in the dataset; only different QSM reconstruction pipelines were used to process them.

^†^ Actual value = 0.001.

### Biologic Findings

**Figure 6:**
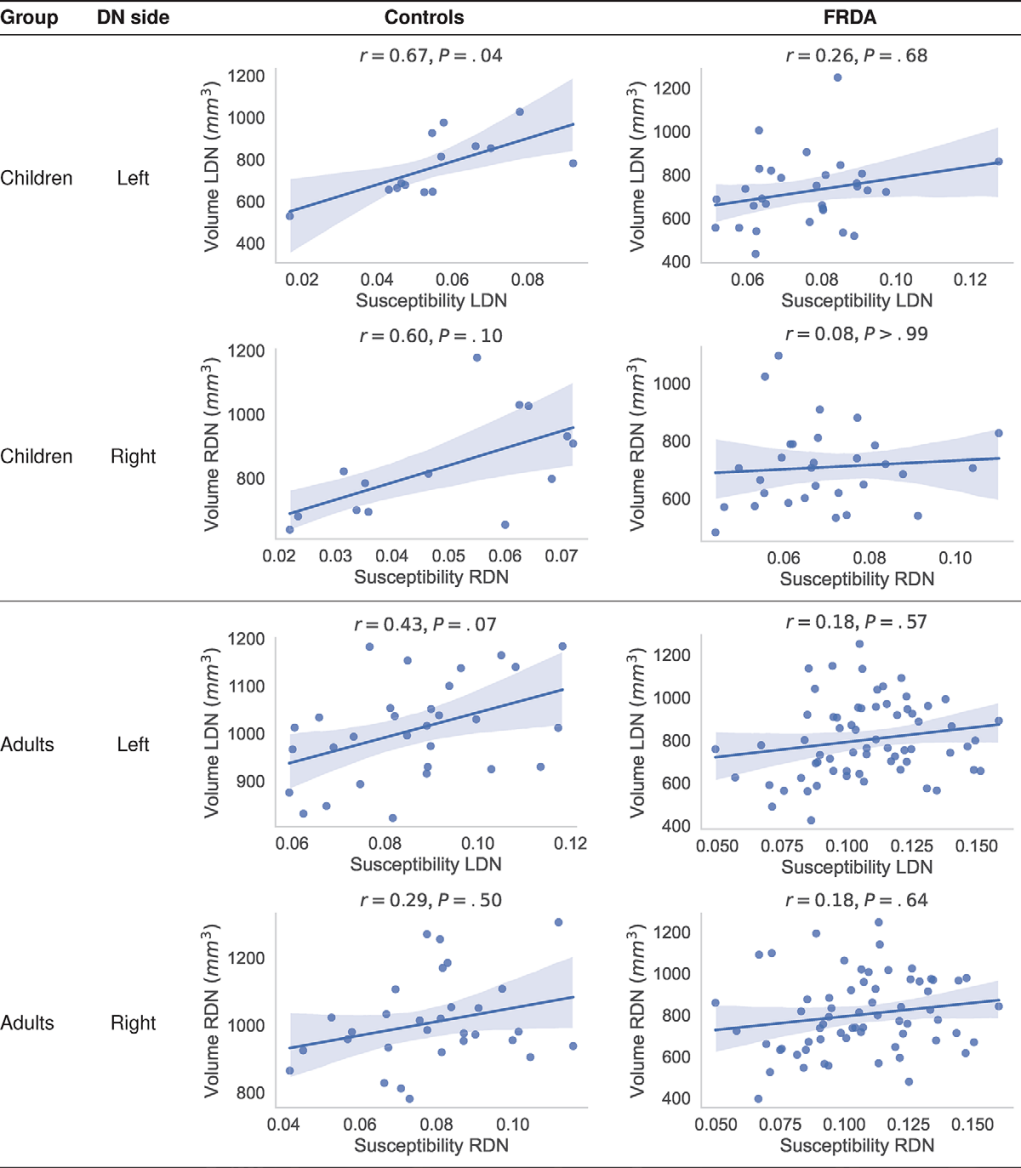
Graphs show the model prediction results. Pearson correlation coefficients and *P* values for each group of individuals and dentate nucleus (DN) side are provided. *P* values were Bonferroni corrected for multiple comparisons. *Children* refers to individuals younger than 18 years of age. The volume estimations were corrected for age and head size (estimated total intracranial volume). FRDA = Friedreich ataxia, LDN = left DN, RDN = right DN.

A positive association trend between DN volume and magnetic susceptibility was observed in healthy controls (Figs 6, S5). These relationships were consistently noted in manual tracings (Fig S5) and model-predicted segmentations (Fig 6) with correction for age and head size (total intracranial volume). This pattern was stronger in children (<18 years of age) than in adults.

This finding suggests that the susceptibility/intensity level may impact border detection due to partial volume effects, possibly introducing a bias in volume estimation, although this effect persists even after z-score intensity normalization.

The impact of susceptibility variability on volume estimation may be estimated, and corrected for, using susceptibility and site (if applicable) as independent variables in multiple linear regression. A correction factor can be estimated from healthy control samples and applied to individuals with disease to avoid confounds between true disease effects and quantification artifacts.

We estimated a representation correction factor (CF = 3636.84) using data from a large, demographically diverse cohort of healthy individuals (age range, 11–64 years old; male:female ratio, 0.93:1) and several imaging protocols. The correction factor was derived using the equation *y*_adj_ = *y* – CF(*x* – *x*_median_), where *y* is the predicted DN volume, *x* is the median DN susceptibility in parts per million, *x*_median_ is the population estimate of median DN susceptibility across a dataset, and CF is the regression/correction factor. To ensure accurate comparative analyses, this formula must be applied uniformly to both disease and control groups within each study.

## Discussion

We proposed and developed an automated DN segmentation solution consisting of a two-step DL pipeline based on convolutional neural networks. The first step prevents false-positive findings in other iron-rich brain structures. A comprehensive dataset of QSM images and high-quality DN manual tracings was generally larger than those used in similar studies (25). Our results demonstrated better performance of the proposed model compared with MRICloud (mean Dice score in external testing, 0.86 ± 0.04 vs 0.57 ± 0.22 [*P* < .001] [LDN]; 0.84 ± 0.07 vs 0.58 ± 0.24 [*P* < .001] [RDN]), handling images acquired under diverse MRI settings and outperforming a similar study that included only healthy individuals (25). The final trained pipeline performed strongly during external testing, and we have introduced guidance regarding appropriately controlling for the influence of magnetic susceptibility on volume measurements.

For artificial intelligence, testing with external datasets is crucial to assess the generalization capacity of the proposed models (26), although rarely reported (27,28). Our external testing provided strong support for the accuracy and robustness of the model, indicated a low risk of overfitting, and exceeded accuracy of MRICloud across a range of conditions. Regarding reconstruction pipeline metrics, consistent Dice performance metrics were observed regardless of which QSM pipeline and reconstruction method was used. A greater score for MEDI-reconstructed images may derive from the fact that experienced raters were exposed to a majority of QSM images processed using this pipeline.

A combination of several factors contributes to the performance of the proposed model. First, we gathered an extensive dataset that captures the diversity found in real-world data. Additionally, we directly compared multiple DL frameworks to select nnU-Net (17) as the optimal architecture, which incorporates a well-known architecture and a thorough set of data augmentation techniques. Moreover, our findings are aligned with analogous studies, such as the one for TotalSegmentator (29), wherein the nnU-Net stands out as a recognized solution for intricate tasks in the medical imaging domain. Also, our proposed pipeline is a two-step solution, comprising localization followed by segmentation. Hence, we drive the focus of the second model into a restricted region of the brain, mitigating false-positive findings, which were observed in morphometrically similar structures of the basal ganglia when a single-step approach was applied to full-head images. The pipeline runs in less than 60 seconds on CPU-only hardware and 15 seconds at most on GPU-based hardware (Intel Core i7, Nvidia RTX 4070 12 GB).

In terms of biologic findings, we replicated significant correlations between DN volume and magnetic susceptibility measures in the healthy population (30,31). These effects were present both in the manual tracings and the prediction masks obtained using the trained models and could be explained by partial volume effects along the edges of the structure, whereby voxels that include a mix of tissue both inside and outside the DN would be more likely to be identified as being inside the DN in individuals with higher mean susceptibility intensity. The result persisted even after intensity normalization. Li and colleagues (30) also reported this finding, but not in other deep gray matter nuclei. If intensity-induced variability were driving partial volume effects, it would be expected to be more severe in small structures such as the DN and the red nucleus (30).

Regardless of the mechanism (biologic or methodologic) underlying this dependency between susceptibility and volume, it could be seen as an artifact that may be accounted for in DN volume assessments. This is further supported by the observation that because susceptibility increases naturally with age, failure to correct for this dependency results in an apparent positive relationship between DN volume and age in the adult population (ie, apparent DN growth over time) (30,31), which is biologically implausible. Care must also be taken when assessing populations with brain pathology, as the relationship between susceptibility and volume in neurologic conditions may be a mix of true disease effects and the artifact described above. Neurologic diseases, such as FRDA, spinocerebellar ataxia type 1, and multiple system atrophy, are characterized by increased DN susceptibility (2,32), which may lead to underestimation of DN volume loss. We therefore recommend statistically controlling for susceptibility levels when inferring volume effects to account for this artifact. In studies of normative populations, this can be achieved through simple regression (eg, including a predictor of noninterest coding for susceptibility in regression models). For studies in pathologic conditions, a correction factor estimated from normative data can be applied. We have reported a correction factor of 3636.84 for adjusting the total volume of DN, which represents the average of LDN and RDN volumes. However, it is important to note that slightly different correction factors were observed for LDN (3553.72) and RDN (3422.21). Consequently, future studies may select the appropriate correction factor based on their specific data and objective. Additionally, adjustments for other confounding variables, such as the impact of aging on DN volumes, susceptibility, or other measures, can be made either in sequential steps or by integrating all the confounding variables into a multiple regression model.

A limitation of this study was the three QSM reconstruction pipelines that were used for generating the training images. Several QSM pipelines are available, including the possibility of combining a wide range of phase unwrapping, background field removal, and susceptibility mapping reconstruction methods and parameters. However, collecting such a diverse dataset would impose another layer of complexity on the study. As the QSM field continues to mature, it is likely that a narrower range of acquisition and reconstruction protocols will become the norm, reducing variability in this dimension. Notably, recent consensus papers have provided recommendations focused on clinical research in this area (33,34), which might be usefully explored in future investigations. Future development work will also investigate the performance benefit of incorporating a standardized QSM reconstruction procedure into the pipeline, providing an end-to-end framework capable of directly processing phase and magnitude input data. Also, new architectures, such as transformer-based models, may be revisited as larger imaging datasets become available. Furthermore, although the dataset included individuals across nine centers, the representation of ethnicities and races was limited, as this was not a primary criterion for eligibility. Another limitation was the inclusion of individuals exclusively diagnosed with FRDA or MS in the disease group, as well as the imbalance of the subgroups and modestly sized external testing samples. Thus, generalizability to other pathologies and out-of-sample datasets may require further validation. However, we have maximized the likelihood of off-the-shelf generalizability of this model by including a training dataset consisting of both clinical and normative data with broad natural variability in the size and QSM intensity of the DN alongside implementation of robust data augmentation techniques (35). Regardless, future work incorporating a broader set of pathologies with known or hypothesized cerebellar involvement in the training dataset, including late-onset spinocerebellar ataxias, multiple system atrophy, essential tremor, autism spectrum disorder, alcohol toxicity, and acquired brain injuries (eg, stroke) that distort the usual anatomic features of the cerebellum, will be key to maximizing the utility of this tool. Implementation of this model in large-scale multicenter observational studies and clinical trials of cerebellar diseases (eg, FRDA trials where DN size and susceptibility may provide secondary or exploratory outcomes) is encouraged to assess its practical utility. Last, histologic validation will be required to provide a true reference standard for comparing the model segmentations from QSM images with laminar measurements of the DN.

In conclusion, our work provides state-of-the-art performance in automated DN segmentation from in vivo MRI. This outcome offers a valuable tool for characterizing cerebellar neuroanatomy in health and disease, particularly in large-scale longitudinal studies. Our solution holds potential to enable the discovery of imaging biomarkers for monitoring disease progression and evaluating treatment efficacy in cerebellar disorders.
